# (*E*)-4-(4-Hydr­oxy-3-nitro­benzyl­idene­amino)-1,5-dimethyl-2-phenyl-1*H*-pyrazol-3(2*H*)-one

**DOI:** 10.1107/S1600536808030031

**Published:** 2008-09-24

**Authors:** Chun-Niu Zhang, Ming-Hua Yang

**Affiliations:** a Department of Chemistry, Lishui University, Lishui 323000, ZheJiang, People’s Republic of China

## Abstract

In the title compound, C_18_H_16_N_4_O_4_, the dihedral angles between the central pyrazole ring and the pendant substituted and unsubstituted aromatic rings are 4.73 (12) and 44.24 (14)°, respectively. An intra­molecular O—H⋯O hydrogen bond occurs. In the crystal structure, an inter­molecular C—H⋯O inter­action may help to consolidate the packing and a short intra­molecular C—H⋯O contact also occurs.

## Related literature

For selected background literature on Schiff bases, see: Alemi & Shaabani (2000 ▶); Kim & Shin (1999 ▶); Yan *et al.* (2006 ▶); Zheng *et al.* (2006 ▶).
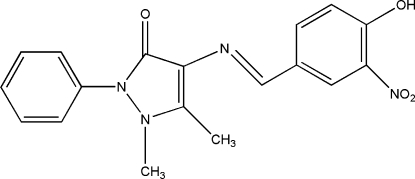


## Experimental

### 

#### Crystal data

C_18_H_16_N_4_O_4_
*M* *_r_* = 352.35
Monoclinic, 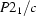

*a* = 7.5000 (15) Å
*b* = 7.8000 (16) Å
*c* = 28.900 (6) Å
β = 95.00 (3)°
*V* = 1684.2 (6) Å^3^
*Z* = 4
Mo *K*α radiation
μ = 0.10 mm^−1^
*T* = 298 (2) K
0.29 × 0.22 × 0.18 mm

#### Data collection

Bruker APEXII CCD diffractometer
Absorption correction: multi-scan (*SADABS*; Sheldrick, 1996 ▶) *T*_min_ = 0.971, *T*_max_ = 0.982
12221 measured reflections
3012 independent reflections
1762 reflections with *I* > 2σ(*I*)
*R*_int_ = 0.063

#### Refinement

*R*[*F* ^2^ > 2σ(*F* ^2^)] = 0.058
*wR*(*F* ^2^) = 0.160
*S* = 0.83
3012 reflections
238 parameters
H-atom parameters constrained
Δρ_max_ = 0.22 e Å^−3^
Δρ_min_ = −0.18 e Å^−3^

### 

Data collection: *SMART* (Bruker, 1998 ▶); cell refinement: *SAINT* (Bruker, 1999 ▶); data reduction: *SAINT*; program(s) used to solve structure: *SHELXS97* (Sheldrick, 2008 ▶); program(s) used to refine structure: *SHELXL97* (Sheldrick, 2008 ▶); molecular graphics: *SHELXTL* (Sheldrick, 2008 ▶); software used to prepare material for publication: *SHELXTL*.

## Supplementary Material

Crystal structure: contains datablocks I, global. DOI: 10.1107/S1600536808030031/hb2795sup1.cif

Structure factors: contains datablocks I. DOI: 10.1107/S1600536808030031/hb2795Isup2.hkl

Additional supplementary materials:  crystallographic information; 3D view; checkCIF report

## Figures and Tables

**Table 1:** Hydrogen-bond geometry (Å, °)

*D*—H⋯*A*	*D*—H	H⋯*A*	*D*⋯*A*	*D*—H⋯*A*
O2—H2⋯O3	0.82	1.88	2.583 (3)	143
C12—H12⋯O1	0.93	2.45	3.096 (3)	127
C18—H18⋯O4^i^	0.93	2.38	3.079 (4)	132

Symmetry code: (i) 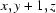.

**Fig. 1.**
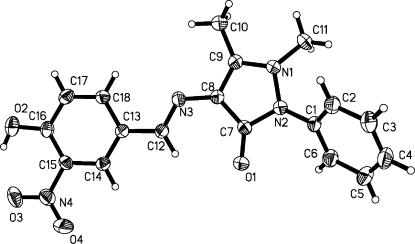
The structure of (I): non-H atoms are shown as 50% probability displacement ellipsoids.

**Fig. 2.**
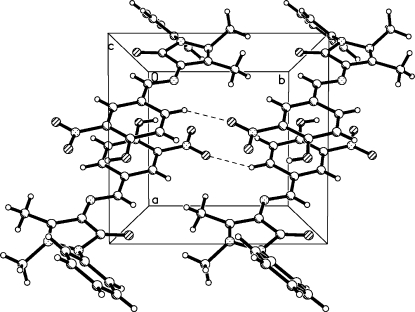
The packing of (I), with C—H···O hydrogen bonds indicated by dotted lines.

## supplementary crystallographic information

### Comment

There have been of great interest in the synthesis, characterization, and properties of Schiff bases and Schiff base complexes. (Yan *et al.*, 2006; Zheng *et al.*, 2006) Schiff bases that have solvent dependent UV/vis spectra (solvatochromicity) can be suitable NLO (nonlinear optical active) materials (Alemi *et al.*, 2000). They are also useful in asymmetric oxidation of methyl phenyl sulfide and enantioselective reactions (Kim *et al.*, 1999).

In this paper, we report here the synthesis and crystal structure of the title compound (I), (Fig. 1). The dihedral angles betweem the pyrazole ring and the pendant C13 and C1 aromatic rings are 4.73 (12)° and 44.24 (14)°, respectively. The C12—N3 bond length of 1.281 (3) Å in (I) is indicative of a normal C=N double bond.

The intra- and intermolecular hydrogen bonds in (I) are listed in Table 1.

### Experimental

Under nitrogen, a mixture of 4-hydroxy-3-nitrobenzaldehyde (1.67 g, 10 mmol) and 4-amino-1,2-dihydro-1,5-dimethyl-1-phenylpyrazol-3-one (2.03 g, 10 mmol) in absolute ethanol (80 ml) was refluxed for about 20 h to yield a yellow precipitate. The product was collected by vacuum filtration and washed with ethanol. The crude solid was redissolved in CH_2_Cl_2_ (70 ml) and washed with water (2 × 8 ml) and brine (10 ml). After being dried over Na_2_SO_4_, the solvent was removed under vacuum, and yellow solid was isolated in a yield of 89% (2.8 g). Colourless blocks of (I) were grown from CH_2_Cl_2_ and absolute ethanol (4:1 v/v) by slow evaporation of the solvent at room temperature over a period of about two weeks.

### Refinement

All the H atoms were placed in calculated positions (C—H = 0.93-0.96Å, O—H = 0.82 Å) and refined as riding, with *U*_iso_(H) = 1.2*U*_eq_(C) or 1.5U_eq_(O, methyl C). The maximum difference peak is located 1.41Å from H11C.

### Crystal data

**Table:**

C_18_H_16_N_4_O_4_	*F*(000) = 736
*M**_r_* = 352.35	*D*_x_ = 1.390 Mg m^−^^3^
Monoclinic, *P*2_1_/*c*	Mo *K*α radiation, λ = 0.71073 Å
Hall symbol: -P 2ybc	Cell parameters from 3012 reflections
*a* = 7.5000 (15) Å	θ = 3.2–25.2°
*b* = 7.8000 (16) Å	µ = 0.10 mm^−^^1^
*c* = 28.900 (6) Å	*T* = 298 K
β = 95.00 (3)°	Block, colourless
*V* = 1684.2 (6) Å^3^	0.29 × 0.22 × 0.18 mm
*Z* = 4	

### Data collection

**Table:**

Bruker APEXII CCD diffractometer	3012 independent reflections
Radiation source: fine-focus sealed tube	1762 reflections with *I* > 2σ(*I*)
graphite	*R*_int_ = 0.063
φ and ω scans	θ_max_ = 25.2°, θ_min_ = 3.2°
Absorption correction: multi-scan (SADABS; Sheldrick, 1996)	*h* = −8→8
*T*_min_ = 0.971, *T*_max_ = 0.982	*k* = −9→9
12221 measured reflections	*l* = −34→34

### Refinement

**Table:**

Refinement on *F*^2^	Primary atom site location: structure-invariant direct methods
Least-squares matrix: full	Secondary atom site location: difference Fourier map
*R*[*F*^2^ > 2σ(*F*^2^)] = 0.058	Hydrogen site location: inferred from neighbouring sites
*wR*(*F*^2^) = 0.160	H-atom parameters constrained
*S* = 0.83	*w* = 1/[σ^2^(*F*_o_^2^) + (0.1*P*)^2^ + 0.3*P*] where *P* = (*F*_o_^2^ + 2*F*_c_^2^)/3
3012 reflections	(Δ/σ)_max_ < 0.001
238 parameters	Δρ_max_ = 0.22 e Å^−^^3^
0 restraints	Δρ_min_ = −0.18 e Å^−^^3^

### Special details

**Table:**

Geometry. All e.s.d.'s (except the e.s.d. in the dihedral angle between two l.s. planes) are estimated using the full covariance matrix. The cell e.s.d.'s are taken into account individually in the estimation of e.s.d.'s in distances, angles and torsion angles; correlations between e.s.d.'s in cell parameters are only used when they are defined by crystal symmetry. An approximate (isotropic) treatment of cell e.s.d.'s is used for estimating e.s.d.'s involving l.s. planes.
Refinement. Refinement of *F*^2^ against ALL reflections. The weighted *R*-factor *wR* and goodness of fit *S* are based on *F*^2^, conventional *R*-factors *R* are based on *F*, with *F* set to zero for negative *F*^2^. The threshold expression of *F*^2^ > σ(*F*^2^) is used only for calculating *R*-factors(gt) *etc*. and is not relevant to the choice of reflections for refinement. *R*-factors based on *F*^2^ are statistically about twice as large as those based on *F*, and *R*- factors based on ALL data will be even larger.

### Fractional atomic coordinates and isotropic or equivalent isotropic displacement parameters (Å^2^)

**Table:**

	*x*	*y*	*z*	*U*_iso_*/*U*_eq_	
N3	0.1353 (3)	0.2354 (3)	0.45196 (7)	0.0455 (5)	
N1	−0.0863 (3)	0.4373 (3)	0.35252 (7)	0.0467 (5)	
N2	−0.1349 (3)	0.2664 (3)	0.34216 (7)	0.0476 (5)	
O1	−0.0566 (2)	−0.0019 (2)	0.37369 (6)	0.0537 (5)	
O2	0.6074 (3)	−0.0082 (3)	0.63167 (6)	0.0704 (6)	
H2	0.6225	−0.1096	0.6384	0.106*	
O3	0.5569 (3)	−0.3329 (3)	0.61887 (8)	0.0807 (7)	
O4	0.3986 (4)	−0.4244 (3)	0.55889 (10)	0.1126 (10)	
N4	0.4606 (3)	−0.3063 (3)	0.58221 (9)	0.0648 (7)	
C13	0.2714 (3)	0.0531 (3)	0.51068 (8)	0.0399 (6)	
C12	0.1594 (3)	0.0821 (3)	0.46713 (8)	0.0429 (6)	
H12	0.1064	−0.0099	0.4507	0.052*	
C8	0.0330 (3)	0.2717 (3)	0.41051 (8)	0.0416 (6)	
C9	0.0055 (3)	0.4366 (3)	0.39532 (8)	0.0459 (6)	
C15	0.4212 (3)	−0.1337 (3)	0.56753 (8)	0.0438 (6)	
C14	0.3086 (3)	−0.1094 (3)	0.52690 (8)	0.0441 (6)	
H14	0.2588	−0.2037	0.5108	0.053*	
C16	0.4969 (3)	0.0046 (4)	0.59269 (8)	0.0475 (6)	
C1	−0.1830 (3)	0.2178 (3)	0.29503 (8)	0.0452 (6)	
C18	0.3488 (3)	0.1923 (3)	0.53588 (8)	0.0469 (6)	
H18	0.3255	0.3032	0.5252	0.056*	
C17	0.4577 (3)	0.1682 (4)	0.57589 (9)	0.0532 (7)	
H17	0.5060	0.2629	0.5920	0.064*	
C5	−0.3461 (4)	0.0338 (4)	0.24047 (11)	0.0689 (9)	
H5	−0.4215	−0.0597	0.2344	0.083*	
C11	−0.2133 (4)	0.5723 (4)	0.33705 (10)	0.0596 (7)	
H11A	−0.3240	0.5546	0.3507	0.089*	
H11B	−0.2346	0.5690	0.3038	0.089*	
H11C	−0.1646	0.6820	0.3465	0.089*	
C6	−0.2976 (3)	0.0829 (4)	0.28630 (9)	0.0567 (7)	
H6	−0.3430	0.0243	0.3107	0.068*	
C7	−0.0519 (3)	0.1560 (3)	0.37629 (8)	0.0426 (6)	
C2	−0.1161 (3)	0.3056 (4)	0.25858 (9)	0.0541 (7)	
H2A	−0.0362	0.3958	0.2644	0.065*	
C10	0.0618 (4)	0.5984 (4)	0.41979 (10)	0.0609 (8)	
H10A	0.1012	0.6792	0.3978	0.091*	
H10B	0.1580	0.5747	0.4430	0.091*	
H10C	−0.0376	0.6456	0.4343	0.091*	
C4	−0.2831 (4)	0.1223 (5)	0.20437 (10)	0.0719 (9)	
H4A	−0.3176	0.0907	0.1739	0.086*	
C3	−0.1694 (4)	0.2575 (4)	0.21333 (10)	0.0647 (8)	
H3A	−0.1273	0.3179	0.1888	0.078*	

### Atomic displacement parameters (Å^2^)

**Table:**

	*U*^11^	*U*^22^	*U*^33^	*U*^12^	*U*^13^	*U*^23^
N3	0.0446 (11)	0.0477 (13)	0.0434 (11)	0.0035 (10)	−0.0014 (9)	0.0012 (10)
N1	0.0526 (12)	0.0365 (12)	0.0494 (12)	0.0018 (9)	−0.0047 (10)	0.0019 (10)
N2	0.0552 (12)	0.0400 (12)	0.0460 (12)	0.0004 (10)	−0.0053 (10)	−0.0005 (10)
O1	0.0650 (11)	0.0391 (12)	0.0553 (11)	0.0023 (9)	−0.0039 (9)	−0.0010 (8)
O2	0.0653 (12)	0.0933 (17)	0.0495 (11)	0.0028 (12)	−0.0130 (9)	−0.0004 (11)
O3	0.0720 (13)	0.0872 (17)	0.0802 (15)	0.0107 (12)	−0.0085 (12)	0.0363 (13)
O4	0.156 (3)	0.0433 (14)	0.128 (2)	0.0001 (15)	−0.046 (2)	0.0050 (15)
N4	0.0638 (15)	0.0563 (17)	0.0732 (17)	0.0019 (13)	−0.0001 (13)	0.0142 (14)
C13	0.0383 (12)	0.0411 (15)	0.0407 (13)	−0.0002 (11)	0.0055 (10)	−0.0025 (11)
C12	0.0407 (13)	0.0445 (15)	0.0432 (13)	−0.0011 (11)	0.0014 (11)	−0.0032 (12)
C8	0.0411 (13)	0.0407 (15)	0.0425 (13)	0.0012 (11)	0.0003 (11)	−0.0008 (11)
C9	0.0463 (13)	0.0438 (15)	0.0470 (14)	0.0001 (12)	0.0008 (12)	−0.0019 (12)
C15	0.0439 (13)	0.0415 (15)	0.0462 (14)	0.0022 (11)	0.0057 (11)	0.0083 (11)
C14	0.0429 (13)	0.0420 (15)	0.0474 (14)	−0.0048 (11)	0.0038 (11)	−0.0009 (11)
C16	0.0443 (13)	0.0605 (17)	0.0374 (12)	0.0003 (13)	0.0020 (11)	0.0000 (12)
C1	0.0433 (13)	0.0477 (15)	0.0429 (14)	0.0014 (12)	−0.0057 (11)	−0.0020 (12)
C18	0.0521 (14)	0.0410 (15)	0.0471 (14)	0.0015 (12)	0.0019 (12)	−0.0029 (12)
C17	0.0552 (15)	0.0544 (17)	0.0495 (15)	−0.0047 (13)	0.0017 (12)	−0.0136 (13)
C5	0.0616 (18)	0.072 (2)	0.0689 (19)	−0.0080 (16)	−0.0160 (16)	−0.0100 (17)
C11	0.0629 (17)	0.0473 (17)	0.0666 (18)	0.0118 (14)	−0.0056 (14)	0.0077 (14)
C6	0.0508 (15)	0.0625 (18)	0.0556 (16)	−0.0043 (14)	−0.0018 (13)	0.0015 (14)
C7	0.0412 (13)	0.0418 (16)	0.0446 (14)	0.0027 (11)	0.0031 (11)	0.0029 (11)
C2	0.0522 (15)	0.0580 (18)	0.0510 (15)	−0.0007 (13)	−0.0020 (12)	0.0010 (13)
C10	0.0699 (18)	0.0448 (17)	0.0655 (18)	0.0009 (14)	−0.0083 (15)	−0.0039 (14)
C4	0.078 (2)	0.084 (2)	0.0497 (17)	0.0033 (19)	−0.0156 (16)	−0.0075 (16)
C3	0.0688 (18)	0.078 (2)	0.0468 (16)	0.0034 (17)	−0.0002 (14)	0.0073 (15)

### Geometric parameters (Å, °)

**Table:**

N3—C12	1.281 (3)	C14—H14	0.9300
N3—C8	1.394 (3)	C16—C17	1.388 (4)
N1—C9	1.362 (3)	C1—C6	1.368 (4)
N1—N2	1.407 (3)	C1—C2	1.387 (4)
N1—C11	1.464 (3)	C18—C17	1.369 (3)
N2—C7	1.412 (3)	C18—H18	0.9300
N2—C1	1.429 (3)	C17—H17	0.9300
O1—C7	1.235 (3)	C5—C4	1.369 (4)
O2—C16	1.343 (3)	C5—C6	1.396 (4)
O2—H2	0.8200	C5—H5	0.9300
O3—N4	1.246 (3)	C11—H11A	0.9600
O4—N4	1.210 (3)	C11—H11B	0.9600
N4—C15	1.435 (3)	C11—H11C	0.9600
C13—C14	1.372 (3)	C6—H6	0.9300
C13—C18	1.404 (3)	C2—C3	1.385 (4)
C13—C12	1.469 (3)	C2—H2A	0.9300
C12—H12	0.9300	C10—H10A	0.9600
C8—C9	1.369 (3)	C10—H10B	0.9600
C8—C7	1.445 (3)	C10—H10C	0.9600
C9—C10	1.490 (4)	C4—C3	1.366 (4)
C15—C16	1.394 (4)	C4—H4A	0.9300
C15—C14	1.398 (3)	C3—H3A	0.9300
			
C12—N3—C8	122.3 (2)	C17—C18—H18	119.3
C9—N1—N2	106.88 (19)	C13—C18—H18	119.3
C9—N1—C11	123.0 (2)	C18—C17—C16	121.0 (2)
N2—N1—C11	117.86 (19)	C18—C17—H17	119.5
N1—N2—C7	109.80 (19)	C16—C17—H17	119.5
N1—N2—C1	119.56 (19)	C4—C5—C6	120.4 (3)
C7—N2—C1	124.3 (2)	C4—C5—H5	119.8
C16—O2—H2	109.5	C6—C5—H5	119.8
O4—N4—O3	120.8 (3)	N1—C11—H11A	109.5
O4—N4—C15	119.4 (2)	N1—C11—H11B	109.5
O3—N4—C15	119.8 (3)	H11A—C11—H11B	109.5
C14—C13—C18	118.4 (2)	N1—C11—H11C	109.5
C14—C13—C12	121.3 (2)	H11A—C11—H11C	109.5
C18—C13—C12	120.3 (2)	H11B—C11—H11C	109.5
N3—C12—C13	119.4 (2)	C1—C6—C5	119.6 (3)
N3—C12—H12	120.3	C1—C6—H6	120.2
C13—C12—H12	120.3	C5—C6—H6	120.2
C9—C8—N3	121.5 (2)	O1—C7—N2	123.9 (2)
C9—C8—C7	108.8 (2)	O1—C7—C8	132.3 (2)
N3—C8—C7	129.6 (2)	N2—C7—C8	103.8 (2)
N1—C9—C8	110.2 (2)	C3—C2—C1	119.3 (3)
N1—C9—C10	121.8 (2)	C3—C2—H2A	120.3
C8—C9—C10	128.0 (2)	C1—C2—H2A	120.3
C16—C15—C14	121.5 (2)	C9—C10—H10A	109.5
C16—C15—N4	120.5 (2)	C9—C10—H10B	109.5
C14—C15—N4	118.0 (2)	H10A—C10—H10B	109.5
C13—C14—C15	120.1 (2)	C9—C10—H10C	109.5
C13—C14—H14	119.9	H10A—C10—H10C	109.5
C15—C14—H14	119.9	H10B—C10—H10C	109.5
O2—C16—C17	117.3 (2)	C3—C4—C5	119.7 (3)
O2—C16—C15	125.0 (2)	C3—C4—H4A	120.1
C17—C16—C15	117.7 (2)	C5—C4—H4A	120.1
C6—C1—C2	120.2 (2)	C4—C3—C2	120.8 (3)
C6—C1—N2	118.8 (2)	C4—C3—H3A	119.6
C2—C1—N2	121.0 (2)	C2—C3—H3A	119.6
C17—C18—C13	121.4 (2)		

### Hydrogen-bond geometry (Å, °)

**Table:**

*D*—H···*A*	*D*—H	H···*A*	*D*···*A*	*D*—H···*A*
O2—H2···O3	0.82	1.88	2.583 (3)	143
C12—H12···O1	0.93	2.45	3.096 (3)	127
C18—H18···O4^i^	0.93	2.38	3.079 (4)	132

Symmetry codes: (i) *x*, *y*+1, *z*.
